# The model cyanobacteria *Anabaena* sp. PCC 7120 possess an intact but partially degenerated gene cluster encoding gas vesicles

**DOI:** 10.1186/s12866-020-01805-8

**Published:** 2020-05-06

**Authors:** Kun Cai, Bo-Ying Xu, Yong-Liang Jiang, Ying Wang, Yuxing Chen, Cong-Zhao Zhou, Qiong Li

**Affiliations:** 1 Hefei National Laboratory for Physical Sciences at the Microscale and School of Life Sciences, University of Science and Technology of China, Hefei, 230027 Anhui China; 2 College of Life Sciences, Chongqing Normal University, Chongqing, 401331 China

**Keywords:** Gas vesicle, Cyanobacteria, Natural isolate, Heterologous expression, Crystal structure, ATPase activity

## Abstract

**Background:**

Bacterial gas vesicles, composed of two major gas vesicle proteins and filled with gas, are a unique class of intracellular bubble-like nanostructures. They provide buoyancy for cells, and thus play an essential role in the growth and survival of aquatic and soil microbes. Moreover, the gas vesicle could be applied to multimodal and noninvasive biological imaging as a potential nanoscale contrast agent. To date, cylinder-shaped gas vesicles have been found in several strains of cyanobacteria. However, whether the functional gas vesicles could be produced in the model filamentous cyanobacteria *Anabaena* sp. PCC 7120 remains controversial.

**Results:**

In this study, we found that an intact *gvp* gene cluster indeed exists in the model filamentous cyanobacteria *Anabaena* sp. PCC 7120. Real-time PCR assays showed that the *gvpA* gene is constitutively transcribed in vivo, and its expression level is upregulated at low light intensity and/or high growth temperature. Functional expression of this intact *gvp* gene cluster enables the recombinant *Escherichia coli* to gain the capability of floatation in the liquid medium, thanks to the assembly of irregular gas vesicles. Furthermore, crystal structure of GvpF in combination with enzymatic activity assays of GvpN suggested that these two auxiliary proteins of gas vesicle are structurally and enzymatically conserved, respectively.

**Conclusions:**

Our findings show that the laboratory strain of model filamentous cyanobacteria *Anabaena* sp. PCC 7120 possesses an intact but partially degenerated gas vesicle gene cluster, indicating that the natural isolate might be able to produce gas vesicles under some given environmental stimuli for better floatation.

## Background

Gas vesicles (GVs), a unique class of intracellular bubble-like nanostructures, are found in many aquatic and soil microbes including halophilic archaea, photosynthetic bacteria, and heterotrophic bacteria [1]. Ambient gases could freely diffuse into and out of GVs, whereas water is impermeable, making the GV a gas-filled organelle [2, 3]. GVs could regulate the buoyancy of microbial cells, enabling the vertical floatation to an appropriate depth in aqueous environments for a better access of oxygen, light and even nutrients [4]. As an organelle composed of only proteins, GV adopts a spindle-shaped cylinder with conical end caps, usually of 45 ~ 250 nm in width and 100 ~ 2000 nm in length [5]. The unique physical properties allow GVs to serve as a potential nanoscale contrast agent for ultrasound and magnetic resonance imaging, which yields multimodal and noninvasive biological imaging with high spatial and temporal resolution [6].

As previously reported, formation of GVs is related to a conserved cluster of 8 ~ 14 genes (termed gas vesicle protein gene cluster, or *gvp* gene cluster for short), encoding two major structural proteins and several essential minor components that might putatively function as chaperones, nucleators and regulators [2, 5, 7]. The primary structural protein GvpA and the external scaffold protein GvpC constitute the 2-nm-thick outer amphiphilic shell of the GV [2, 5, 8]. GvpA, a 7.5-kDa highly conserved and hydrophobic protein, assembles into tandem arrays that form 4.6-nm-wide characteristic ribs running nearly perpendicular to the long axis of the GV [9, 10]. Notably, most cyanobacteria possess multiple copies of *gvpA* gene, for example, two in *Calothrix* sp. [11], three in *Microcystis aeruginosa* [12] and five in *Anabaena flos-aquae* [13]. In contrast, GvpC is a less-abundant, not conserved, and highly hydrophilic protein [14]. GvpC usually contains a number of conserved 33-residue repeating motif (33RR), and functions to connect GvpA molecules in the same and/or adjacent ribs to strengthen and stabilize the shell of GV [15]. In vitro experiments demonstrated that removal of GvpC leads to a three-fold decrease of the critical collapse pressure of GVs, whereas addition of GvpC helps GVs to restore normal strength [16, 17]. In addition, GvpF is reported to be a structural protein localized at the inner surface of GVs [18].

To date, a series of cyanobacteria have been found to produce GVs, such as *A. flos-aquae*, *Calothrix* sp. PCC 7601, *M. aeruginosa* PCC 7806, *Oscillatoria* sp. 6412, *Pseudanabaena*, *Nostoc* sp. 6705 [12, 19]. Notably, filamentous cyanobacteria *Calothrix* and *Nostoc* can differentiate hormogonia upon environmental stimuli, the process of which is characterized by the formation of GVs [2, 20]. Despite the laboratory strain of model filamentous cyanobacteria *Anabaena* sp. PCC 7120 fails in differentiating hormogonia [19, 21], it remains unknown whether the natural isolate could differentiate hormogonia and produce GVs. Here we found that *Anabaena* sp. PCC 7120 possesses an intact *gvp* gene cluster, which shares an organization similar to that of previously identified GV-forming cyanobacteria. The results of real-time PCR showed that *gvpA* is constitutively transcribed in vivo, and its expression level could be augmented at an altered light intensity and growth temperature. The complete *gvp* gene cluster could be heterologously expressed and assembled into irregular GVs in *Escherichia coli*. Moreover, structural combined with enzymatic investigations suggested that GvpF and GvpN are structurally and enzymatically conserved, respectively. These findings indicated that the natural isolate of *Anabaena* sp. PCC 7120 is most likely able to produce GVs under some given environmental stimuli.

## Results

### Organization and conservation of the *gvp* genes in *Anabaena* sp. PCC 7120

**Fig. 1:**
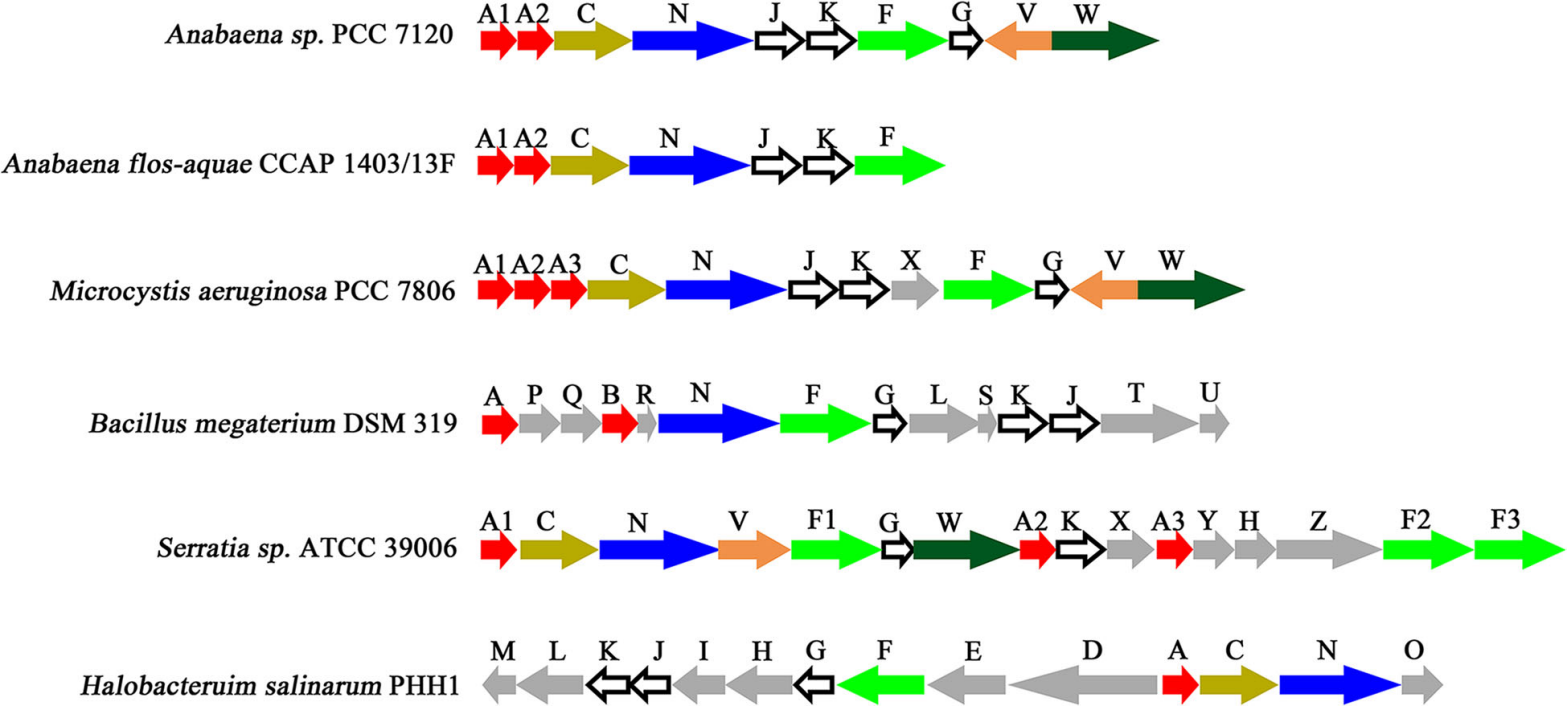
Organizations of the *gvp* gene cluster from different species of bacteria. Each alphabet above the arrow represents a *gvp* gene. Transcription direction of each gene is indicated by the arrow. The *gvp* genes absent in *Anabaena* sp. PCC 7120 are shown as grey arrows

The entire genomic sequence of the model filamentous nitrogen-fixing cyanobacteria *Anabaena* sp. PCC 7120 was reported in 2001, which consists of a single circular genome of 6,413,771 bp and six plasmids [22]. Eight out of the 5368 putative open reading frames in the genome were annotated as *gvp* genes: *gvpA*, *gvpB*, *gvpC*, *gvpN*, *gvpJ*, *gvpK*, *gvpF* and *gvpG*, without annotations of *gvpV* and *gvpW* compared to some other *gvp* gene clusters. Using BlastP program, we found that the proteins encoded by *alr2246* and *alr2245*, two genes at the downstream of *gvpG*, share a sequence similarity of 62% and 65% to GvpV and GvpW of *M. aeruginosa* PCC 7806, respectively. Thus we assigned *alr2246* and *alr2245* to *gvpV* and *gvpW*, respectively (Fig. 1). It suggested that *Anabaena* sp. PCC 7120 possesses an intact *gvp* gene cluster, which shares a gene organization similar to that in the previously reported GV-forming cyanobacteria, such as *A. flos-aquae* and *M. aeruginosa* PCC 7806. Notably, most of the *gvp* genes in GV-forming *Haloarchaea* and other bacteria are highly conserved [5], despite the gene organizations vary a lot (Fig. 1).

**Fig. 2:**
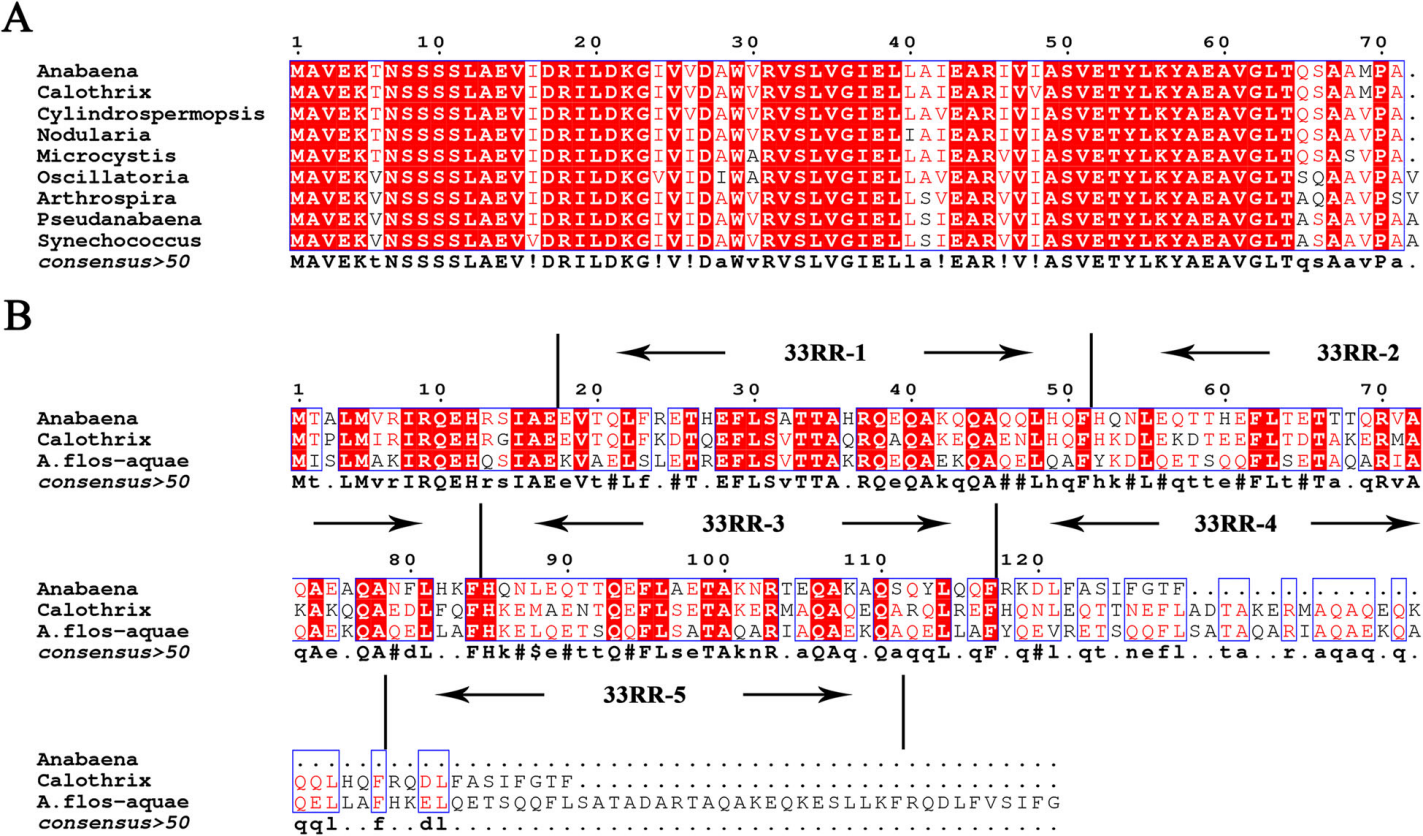
Conservativity of GvpA and GvpC. **a** Multiple-sequence alignment of GvpA from different cyanobacterial strains. The alignment was performed with the program Multalin. All sequences were downloaded from the NCBI database (www.ncbi.nlm.nih.gov) with the following accession numbers: *Anabaena* sp. PCC 7120, WP_010996411; *Calothrix* sp. PCC 7103, WP_011316976; *Nodularia spumigena* CCY9414, AHJ27872; *Microcystis aeruginosa* PCC 7806, WP_084989880; *Cylindrospermopsis raciborskii*, WP_057178839; *Oscillatoria* sp. PCC 10802, WP_017721733; *Arthrospira platensis*, *WP_006616598*; *Pseudanabaena* sp. SR411, WP_009626980; *Synechococcus* sp. PCC 7502, WP_015167036. **b** Multiple-sequence alignment shows the 33RRs of GvpC. All sequences were downloaded from the NCBI database with the following accession numbers: *Anabaena* sp. PCC 7120, WP_010996410; *A. flos-aquae*, AAA58710; *Calothrix* sp. PCC 7103, EKF01074

Multiple-sequence alignment showed that *gvpB* is nearly identical to *gvpA* in the *gvp* gene cluster of *Anabaena* sp. PCC 7120, suggesting that *gvpB* is an isoform of *gvpA*. Accordingly, *gvpA* and *gvpB* should be re-annotated to *gvpA1* and *gvpA2*, respectively (Fig. 1). Moreover, GvpA of *Anabaena* sp. PCC 7120 shares a sequence similarity to those of other cyanobacterial strains up to 90% (Fig. 2a), indicating that the primary structural protein GvpA exhibits a rarely high conservativity in cyanobacteria. Further sequence analysis revealed that the external scaffold protein GvpC of *Anabaena* sp. PCC 7120 contains only three conserved 33RRs (Fig. 2b), which probably result in GVs of smaller diameter. In fact, a previous report revealed that *A. flos-aquae* GVs with a GvpC of five 33RRs have a larger diameter compared to those of *Calothrix* sp. PCC 7601 with a GvpC of four 33RRs [23].

### The *gvpA* gene is upregulated at low light intensity and high temperature

**Fig. 3:**
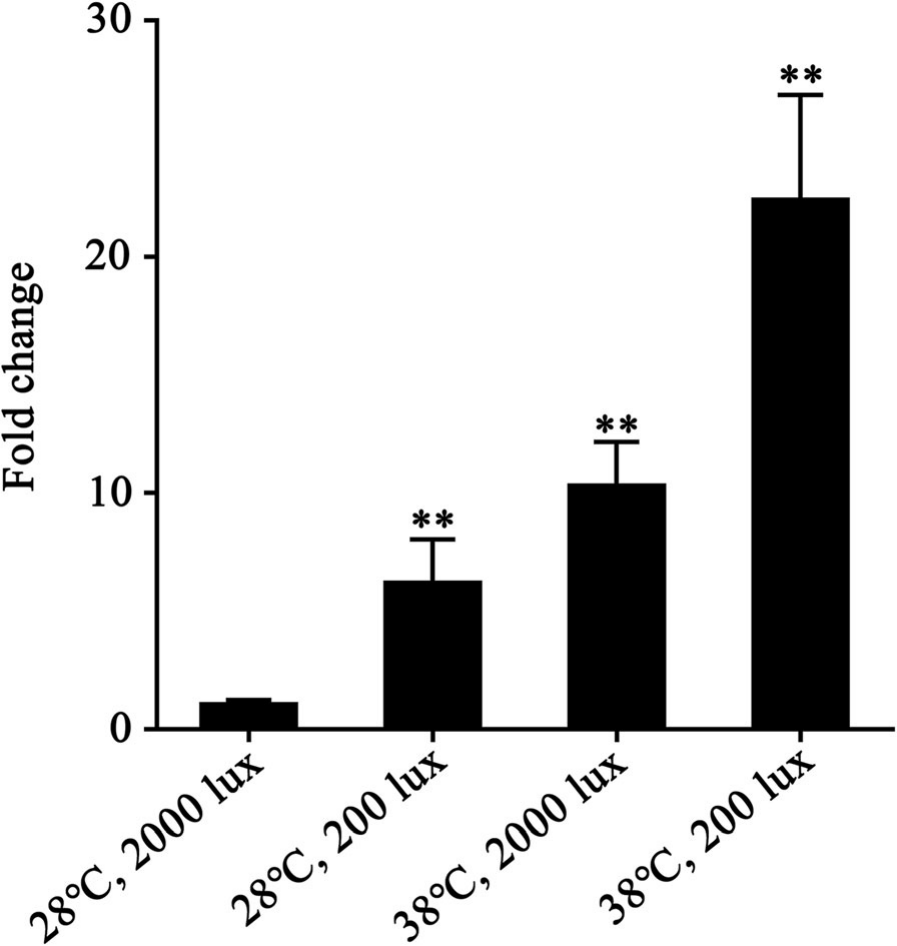
Expression levels of *gvpA* under different growth conditions detected by real-time PCR. Each histogram represents the average value of triplicate experiments, and a two-tailed Student’s *t* test was used for the comparison of statistical significance (^**^P < 0.01)

Considering that the transcription of *gvp* genes is the prerequisite of GV formation, we investigated whether the *gvpA* gene, encoding the major structural component of GV, could be transcribed in vivo. The total RNA was extracted from *Anabaena* sp. PCC 7120 cells grown at a light intensity of 2000 lux at 28 °C, and applied to real-time PCR assays. The results showed that an expected fragment of *gvpA* could be detected (Fig. 3), suggesting that the *gvpA* gene is constitutively transcribed in *Anabaena* sp. PCC 7120 under normal laboratory growth condition.

Afterwards, we shifted *Anabaena* sp. PCC 7120 cells to various external stimuli and detected the expression profiles of *gvpA* gene. The expression level of *gvpA* in *Anabaena* sp. PCC 7120 upon a single stimulus of low light intensity at 200 lux or high temperature at 38 °C were elevated to 6 and 11 folds, respectively (Fig. 3), compared to the constitutive expression level. Moreover, when *Anabaena* sp. PCC 7120 cells were grown under the condition of double stimuli of both low light intensity and high temperature, the expression level of *gvpA* was upregulated approximately 23 folds (Fig. 3). Considering that GvpA is the primary structural component of GV, we speculated that prototype GVs could be produced in *Anabaena* sp. PCC 7120 at some given conditions. However, we failed in observing the floatation of *Anabaena* sp. PCC 7120 cells in response to the above double stimuli. It implied that mature and functional GVs do not exist in the laboratory strain of *Anabaena* sp. PCC 7120, in consistence with its incapability of differentiating hormogonia.

### Expression of the *gvp* gene cluster of *Anabaena* sp. PCC 7120 in *E. coli*

**Fig. 4:**
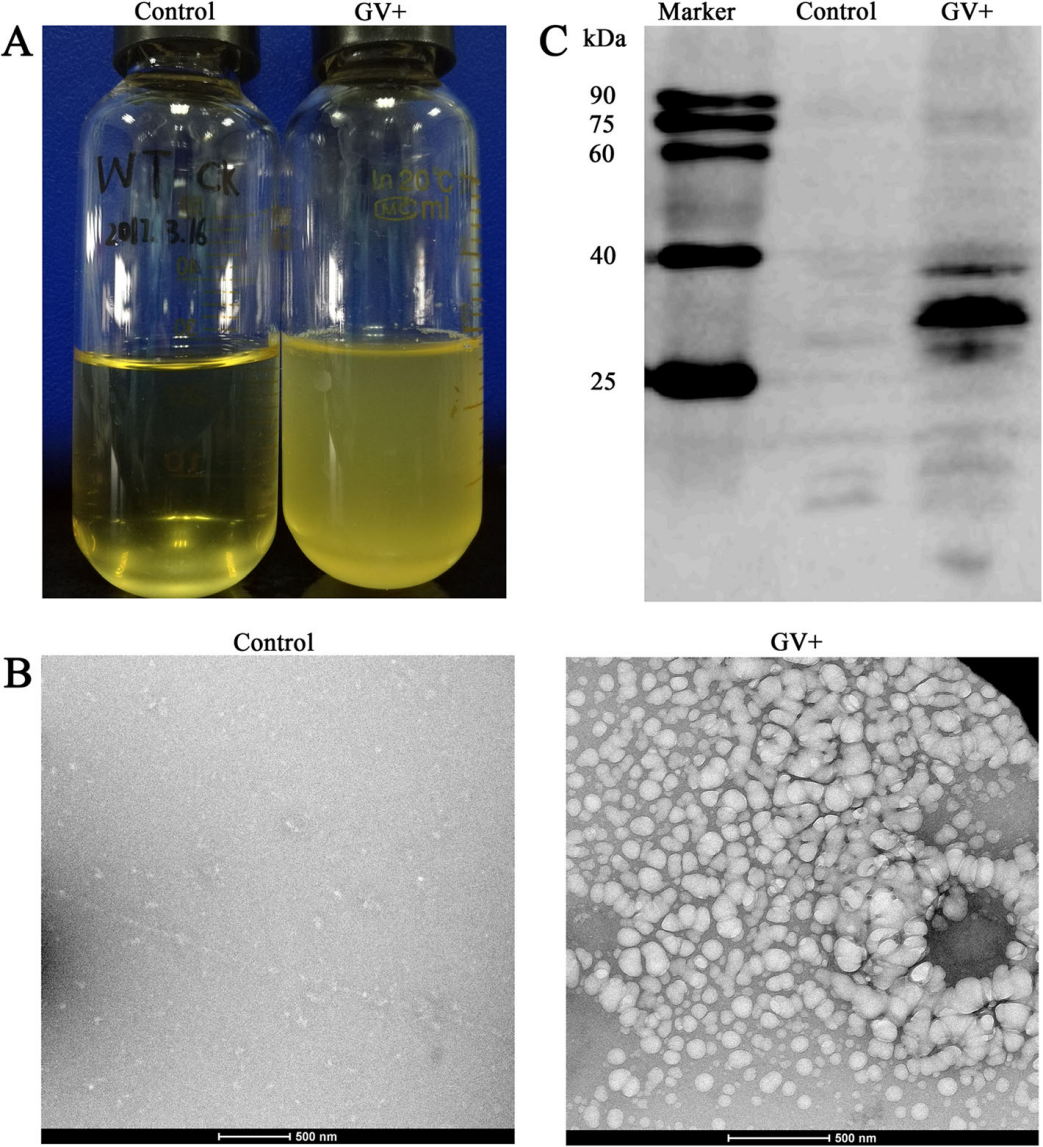
Expression of the *gvp* gene cluster of *Anabaena* sp. PCC 7120 in *E. coli*. **a** Photographs of *E. coli* cells transformed with recombinant *gvp* plasmids and control vectors, respectively. **b** Negative-staining electron microscopy images of the putative gas vesicles purified from *E. coli* cells expressing the *gvp* genes (right) and the control vectors (left). **c** Western blot of the purified gas vesicles. The probe is anti-His antibody. The prestained protein standards are displayed in the lane marked Marker and their molecular masses are indicated in kDa. The lanes marked control and GV+ indicate the control and the experimental group, respectively

The *gvp* genes of *Anabaena* sp. PCC 7120 were constructed in the expression vectors and then transformed to *E. coli* cells. Interestingly, we observed that the *E. coli* cells transformed with the recombinant *gvp* plasmids exhibit a buoyancy phenotype, whereas the cells carrying the control vectors sink to the bottom of the tube (Fig. 4a). Then, the turbidity measurements showed that the upper fraction of the culture medium of the experimental group has an absorbance of 0.57 at 600 nm, compared to 0.02 of the control. The results suggested that GVs might be produced in the recombinant *E. coli* cells that harbor the *gvp* gene cluster of *Anabaena* sp. PCC 7120.

Afterwards, the recombinant GVs were purified from *E. coli* cells and applied to transmission electron microscopy, following a previously reported protocol [18]. The image displayed a large number of round and oval bubble-like structures (Fig. 4b), similar to those irregular GVs via expressing *Bacillus megaterium gvp* gene cluster in *E. coli* [24]. In contrast, similar bubble-like structures were absent from the *E. coli* cells that expressing the control vectors (Fig. 4b). Western blot assays further showed that the purified GVs possess enriched His-tagged GvpA proteins (Fig. 4c). Altogether, transformation of the *gvp* gene cluster of *Anabaena* sp. PCC 7120 enabled *E. coli* to display a buoyancy phenotype, because of the assembly of GVs, indicating that *Anabaena* sp. PCC 7120 possesses an intact *gvp* gene cluster capable of heterogeneously producing irregular but functional GVs.

### The crystal structure of GvpF

**Fig. 5:**
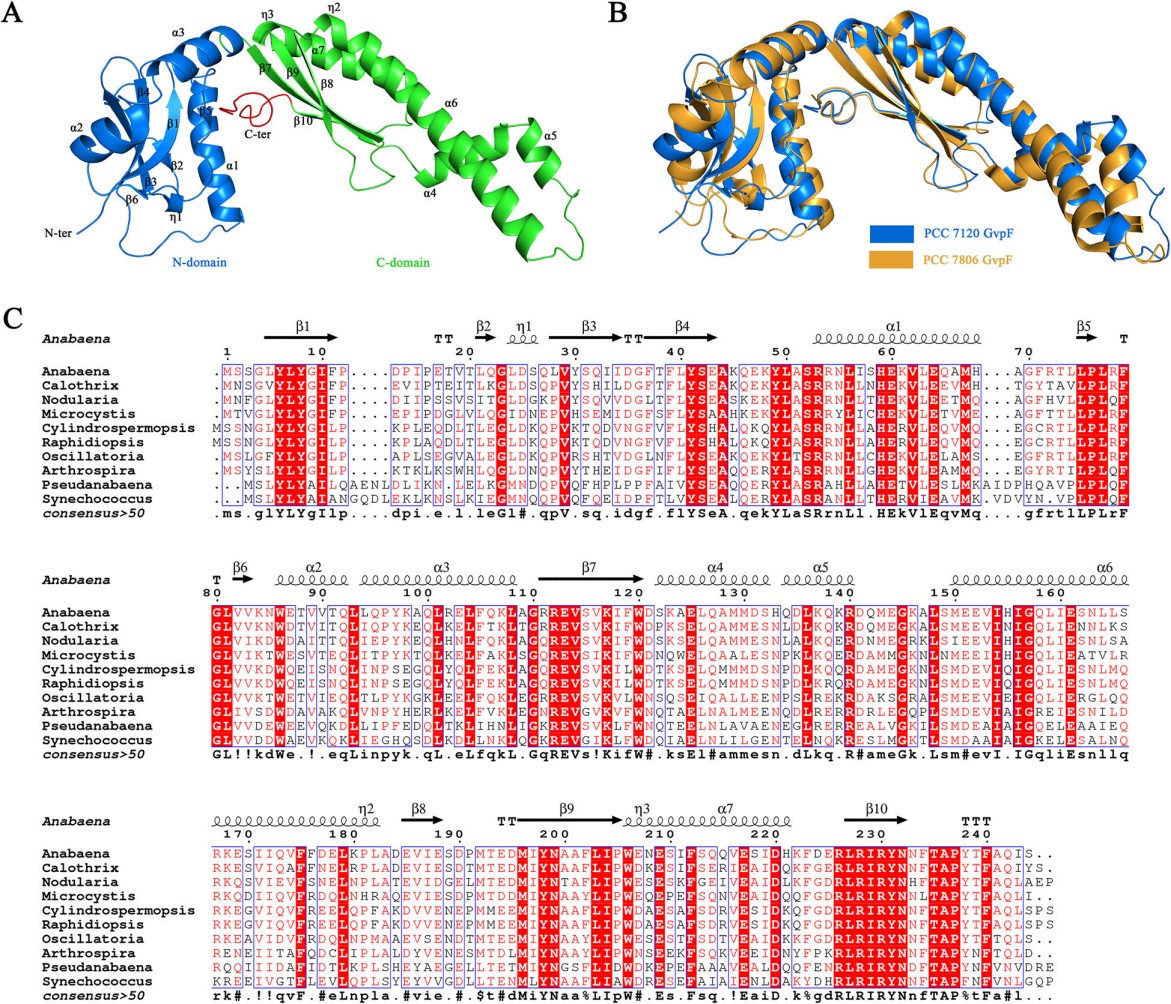
Crystal structure of GvpF. **a** Cartoon representation of the overall structure. The N-domain, C-domain and C-terminal tail are colored in blue, green and red, respectively. The secondary structural elements are labeled sequentially. **b** Structural superposition of *Anabaena* sp. PCC 7120 GvpF (blue) on *M. aeruginosa* PCC 7806 GvpF (orange; PDB ID: 4QSG). **c** Multiple-sequence alignment of GvpF from different cyanobacterial strains. The alignment was performed with the program Multalin. The corresponding secondary structural elements of GvpF are displayed above the sequences. All sequences were downloaded from the NCBI database with the following accession numbers: *Anabaena* sp. PCC 7120, BAB73947; *Calothrix* sp. PCC 7103, WP_019492269; *Nodularia spumigena* CCY9414, EAW43904; *Microcystis aeruginosa* PCC 7806, CAE11906; *Cylindrospermopsis raciborskii*, WP_085729041; *Raphidiopsis brookii* D9, EFA73417; *Oscillatoria* sp. PCC 10802, WP_017721720; *Arthrospira platensis*, WP_006616264; *Pseudanabaena* sp. SR411, WP_094529688; *Synechococcus* sp. PCC 7502, AFY73702

To further investigate the putative GVs of *Anabaena* sp. PCC 7120 from the structural point of view, all Gvp proteins, except for GvpA and GvpC, were successfully overexpressed, purified and applied to crystal screening; however, only the crystal structure of GvpF was eventually solved at 2.55 Å resolution in the space group *C222*_*1*_ (Table 1). *Anabaena* sp. PCC 7120 GvpF is composed of two structurally separated domains, both of which display a fold in α + β class, followed by a C-terminal tail inserted into the middle area of the two domains (Fig. 5a). Further structural analysis showed that the N-domain of GvpF displays an architecture in which a six-stranded β-sheet (β1-β6) is sandwiched by two α-helices (α1-α2) and the helix η1, whereas the C-domain adopts a modified ferredoxin fold owing to an extension region consisting of three consecutive helices (α4, α5 and the N-terminal segment of α6) (Fig. 5a). Moreover, the additional C-terminal tail provides an interface for the N-domain and C-domain to pack against each other, resulting in the structural stability and correct folding of GvpF (Fig. 5a).

**Table 1:** Crystal parameters, data collection, and structure refinement

	GvpF
**Data collection**	
Space group	*C222*_*1*_
Unit cell parameters	
*a, b, c* (Å)	96.40, 147.09, 106.58
*α*, *β*, *γ* (°)	90.00, 90.00, 90.00
Resolution range (Å)	50.00–2.55 (2.62–2.55)^*a*^
Unique reflections	24, 900 (2, 440)
Completeness (%)	99.9 (100)
<*I/σ(I)*>	16.3 (2.9)
*R*_merge_^*b*^ (%)	10.6 (69.1)
Average redundancy	10.9 (10.9)
**Structure refinement**	
Resolution range (Å)	44.46–2.55
*R*_factor_^*c*^/*R*_free_^*d*^ (%)	19.57/25.61
Number of protein atoms	3, 962
Number of water atoms	28
RMSD^*e*^ bond lengths (Å)	0.015
RMSD bond angles (°)	1.709
Mean B factors (Å^2^)	53.189
Ramachandran plot (residues, %)^*f*^	
Most favored	95.80
Allowed	4.00
Outliers	0.20
Protein Data Bank entry	6L5D

^*a*^ Highest resolution shell is shown in parenthesis. R_sym_ = Σ_h_Σ_i_|I_h,i_ − I_h_|/Σ_h_Σ_i_I_h,i_, where *I*_*h*_ is the mean intensity of the *i* observations of symmetry related reflections of *h*. R = Σ|F_obs_ − F_calc_|/ΣF_obs_, where *F*_*obs*_ = *F*_*p*_, and *F*_*calc*_ is the calculated protein structure factor from the atomic model. RMSD in bond lengths and angles are the deviations from ideal values

DALI search [25] revealed that *Anabaena* sp. PCC 7120 GvpF shares a high structural homology to the previously reported GvpF of *M. aeruginosa* PCC 7806 (PDB code: 4QSG, Z score 27.5, sequence identity 67%), with a root-mean-square deviation of 1.8 Å over 238 Cα atoms. Superposition of the two structures showed a very similar structure, except that GvpF of *Anabaena* sp. PCC 7120 possesses a shorter helix α5 in the C-domain (Fig. 5b). Besides, structure-based multiple-sequence alignment revealed that GvpF proteins are highly conserved among diverse species of cyanobacteria (Fig. 5c). It indicated that *Anabaena* sp. PCC 7120 GvpF might also function as a structural protein involved in forming GVs as that of *M. aeruginosa* PCC 7806 [18].

### GvpN is an active ATPase

**Fig. 6:**
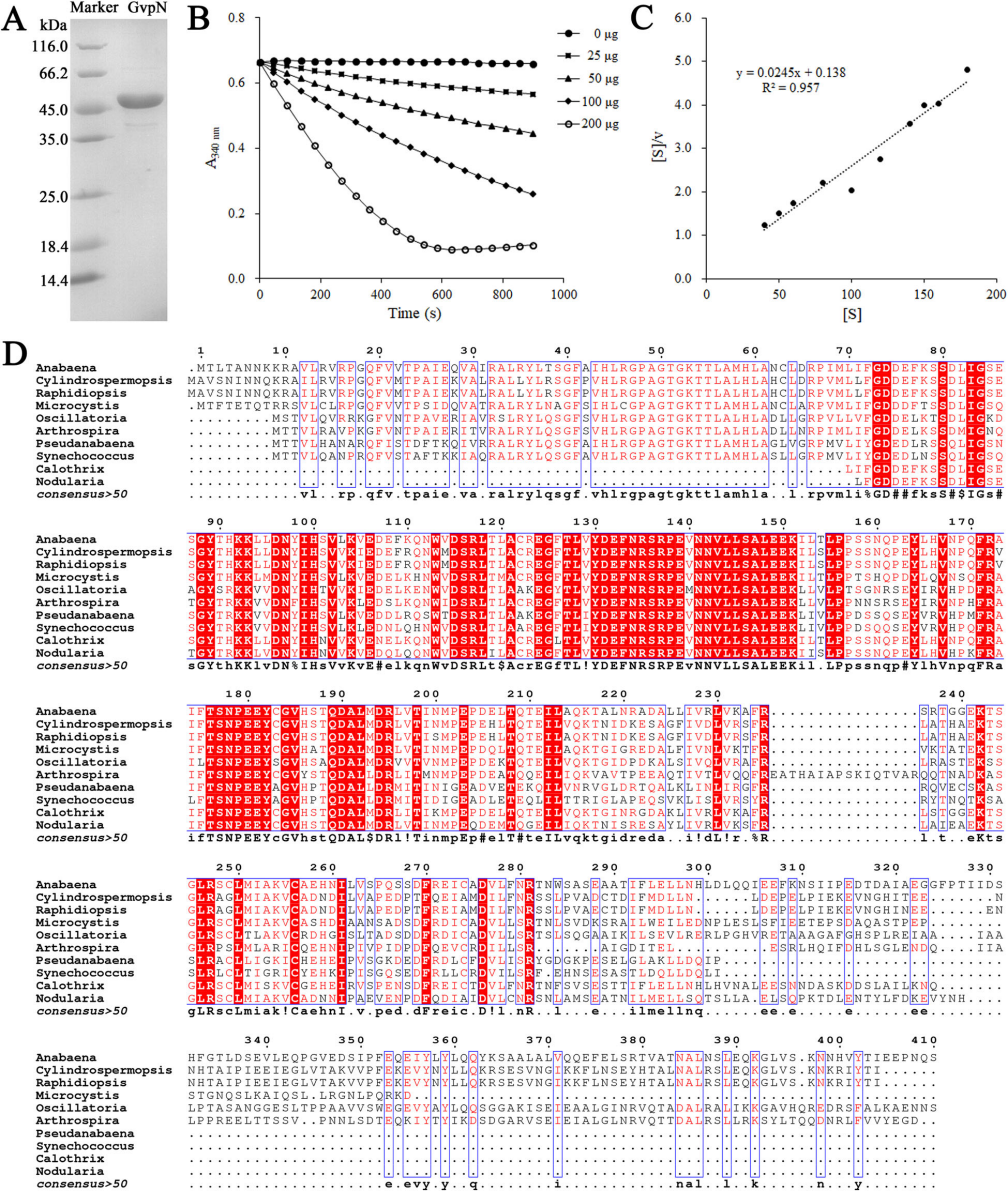
GvpN is an active ATPase. **a** SDS-PAGE of the purified GvpN protein. **b** The enzymatic profiles of GvpN. The final amounts of recombinant GvpN in the 200-μL system are 0, 25, 50, 100 and 200 μg, respectively. The decrease in absorbance at 340 nm was monitored using a DU800 spectrophotometer. **c** The Hanes-Woolf plot of GvpN. **d** Multiple-sequence alignment of GvpN from different species of cyanobacteria. The alignment was performed with the program Multalin. All sequences were downloaded from the NCBI database with the following accession numbers: *Anabaena* sp. PCC 7120, WP_010996409; *Calothrix* sp. PCC 7103, WP_019492266; *Nodularia spumigena* CCY9414, AHJ27875; *Microcystis aeruginosa* PCC 7806, WP_002747926; *Cylindrospermopsis raciborskii*, WP_061547066; *Raphidiopsis brookii* D9, EFA73420; *Oscillatoria* sp. PCC 10802, WP_017715028; *Arthrospira platensis*, WP_006616595; *Pseudanabaena* sp. SR411, WP_094529416; *Synechococcus* sp. PCC 7502, WP_015167038

Sequence analysis against the Pfam database [26] showed that *Anabaena* sp. PCC 7120 GvpN contains an ATPases Associated with various cellular Activities (AAA) domain at the N-terminus, which was previously classified in the AAA+ protein superfamily of the ring-shaped P-loop NTPases [27]. Therefore, the recombinant GvpN of *Anabaena* sp. PCC 7120 was overexpressed in *E. coli* and purified (Fig. 6a), which was applied to the ATPase activity assays. Upon the addition of recombinant GvpN, the substrate ATP was gradually hydrolyzed over time (Fig. 6b). Upon the increase of GvpN added to the reaction, the ATP was hydrolyzed at a higher rate (Fig. 6b), suggesting that GvpN indeed possesses the ATPase activity. Using the Hanes-Woolf plot method (Fig. 6c), we determined the Michaelis-Menten parameters of GvpN towards ATP at a *K*_*m*_ of 3.9 ± 1.5 μM, *k*_*cat*_ of 35 ± 2 s^− 1^ and *k*_*cat*_/*K*_*m*_ of 8.97 s^− 1^ μM^− 1^. Moreover, multiple-sequence alignment showed that the AAA domain of GvpN is highly conserved among different cyanobacterial species (Fig. 6d), indicating that the ATPase activity is a common feature of GvpN. In fact, a previous report showed that deletion of GvpN in *Serratia* sp. ATCC 39006 led to small bicone-shaped GVs and lack of cell buoyancy [28]. All together, it suggested that *Anabaena* sp. PCC 7120 possesses an enzymatically active GvpN, which might be necessary for the formation of mature GVs.

## Discussion

GVs play an essential role in the survival of prokaryotic species, and thus should be assembled or disassembled properly at the right time in response to diverse external stimuli. Actually, the formation of GVs or the expression of genes encoding Gvp proteins are affected by various environmental stimuli, such as temperature, light intensity, oxygen supply, pH and salinity, cell density and carbon source [5, 29–32]. For the cyanobacteria *A. flos-aquae*, *Calothrix* sp. PCC 7601 and *Microcystis* sp. BC 84/1, low light intensity could induce more GVs in order to enable the cells to move towards the surface of the aqueous habitat [5]. Mlouka and colleagues found that lack of nutrition, especially CO_2_ and light irradiance, leads to an augmented production of GVs in *M. aeruginosa* [12]. Moreover, for enterobacteria *Serratia* sp. ATCC39006, the formation of GVs depends on cell density under the control of quorum-sensing signals, and is responsive to oxygen shortage, resulting in facilitating the buoyancy of cells [28, 33]. In addition, for haloarchaea, two regulatory proteins GvpD and GvpE were shown to be involved in regulating the expression of *gvp* genes at both transcriptional and translational level [34]. Altogether, the formation of GVs is necessary for some bacteria in response to various environmental conditions.

The model filamentous and heterocyst-forming cyanobacteria *Anabaena* sp. PCC 7120 were isolated from the Lake Michigan in the late 1960s [22]. Theoretically, the environmental stimuli mentioned above should probably induce the formation of GV in *Anabaena* sp. PCC 7120, which possesses the *gvp* gene cluster. However, no one has observed GVs in the past 50 years of studying the physiology of this model organism. In this study, we found that *Anabaena* sp. PCC 7120 has an intact *gvp* gene cluster similar to those GV-forming cyanobacterial strains (Fig. 1). In fact, *A. flos-aquae* and *M. aeruginosa* PCC 7806 were proved to be able to produce cylinder-shaped GVs, the formation of which was regulated by light intensity [2, 12]. It is most likely that *Anabaena* sp. PCC 7120 is also capable of forming GVs under some given environmental stimuli. Indeed, the primary structural gene *gvpA* of *Anabaena* sp. PCC 7120 is constitutively transcribed, and could be upregulated at low light intensity and high temperature (Fig. 3). Heterologous expression of the intact *Anabaena* sp. PCC 7120 *gvp* gene cluster enabled *E. coli* cells to gain the capacity of floatation, thanks to the formation of irregular GVs (Fig. 4). However, we failed in either setting up a reproducible procedure to enable the floatation of *Anabaena* sp. PCC 7120, or seeing GVs inside the cells, or purifying GVs from this laboratory strain, after extensive trials of various stimuli and combinations. It indicated that the *gvp* gene cluster of *Anabaena* sp. PCC 7120 was partially degenerated in the 50-year laboratory culture.

Notably, formation of GVs is a key feature accompanied with the differentiation of hormogonia, which has already been proved in filamentous cyanobacteria *Calothrix* and *Nostoc* [2, 20]. Recently, Gonzalez and colleagues reported that hormogonia differentiation is regulated by a hierarchal sigma factor cascade in the filamentous cyanobacteria *Nostoc punctiforme*, which retain the developmental complexity of natural isolates [21]. In detail, the sigma factor sigJ activates the expression of both *sigC* and *sigF* genes, as well as other hormogonium-specific genes; meanwhile, sigJ controls the transcription of *gvpA* gene via binding at the − 10 region, which is a consensus sigJ-dependent promoter (designated as J-Box, GGGaAtacT) [21]. However, we found that the highly conserved GGG stretch of J-Box was mutated to AGC at the upstream promoter region of *gvpA* in the laboratory strain of *Anabaena* sp. PCC 7120, which might result in the altered binding affinity towards sigJ, and eventually the failure of producing functional GVs. It indicated that the natural isolate of *Anabaena* sp. PCC 7120, without the mutations at the regulatory region of *gvp* gene cluster, might be capable of differentiating hormogonia and producing GVs for better floatation in response to some given environmental stimuli. More investigations including comparative genomics analyses might help us to clearly elucidate which mutations in the present laboratory strain of *Anabaena* PCC 7120 lead to the loss of function.

## Conclusions

In this study we demonstrated that the laboratory model filamentous cyanobacterium *Anabaena* sp. PCC 7120 indeed possesses an intact but partially degenerated gene cluster encoding gas vesicles, which gives us the hint that its natural isolate was most likely able to produce GVs under some given environmental stimuli. Owing to the fast growth and non-toxicity of the model strain sp. PCC 7120, investigations that enable the large production of GVs in this strain will benefit the potential application of GVs in biological imaging.

## Methods

### RNA extraction and real-time PCR

The *Anabaena* sp. PCC 7120 cells were grown at 28 °C under a light intensity of 2000 lux (supplied from top) with a 12/12 photoperiod in BG-11 medium to an OD_730nm_ of 0.8, and then induced with 200 lux light intensity, 38 °C and both for 24 h, respectively. The stressors were selected according to a previous report summarizing the environmental conditions that could induce the formation of gas vesicles [5]. The cells were harvested by centrifugation and washed twice with the PBS buffer. The total RNA was extracted using the RNeasy Mini Kit (Qiagen, Hilden, Germany) according to the manufacturer’s protocol. The residual genomic DNA was removed by RNase-free DNase (Takara, Shiga, Japan) at 37 °C for 2 h. PCR assays were conducted to confirm the absence of genomic DNA contamination. The RNA quality was checked by agarose gel electrophoresis. The cDNA synthesis was carried out by reverse transcription using the PrimeScript™ RT reagent Kit (Takara, Shiga, Japan).

For real-time PCR, amplification was performed using the FastStart universal SYBR Green Master (Roche, Basel, Switzerland) with the StepOne™ Real-Time PCR System (Applied Biosystems, Carlsbad, USA). The primers for *rnpB* are 5′-GCGATTATCTATCTGGGACG and 5′-CAACTCTTGGTAAGGGTGC, whereas those for *gvpA* are 5′-TGGCAGAAGTTATTGACC and 5′-GAGAAACACGTACCCAAG. Notably, the *rnpB* gene encoding RNaseP subunit B was used as the internal reference gene according to previous real-time PCR experiments concerning cyanobacteria [35]. The PCR conditions were as follows: 1 cycle at 95 °C for 10 min, 40 cycles at 95 °C for 15 s, 60 °C for 60 s, and 72 °C for 20 s; then the melting curve stage was performed rising from 60 °C to 95 °C by every 0.3 °C. The transcription ratios of *gvpA* to *rnpB* were calculated using the relative quantification analysis module of 2^-∆∆Ct^ method based on *Ct* values [36]. All real-time PCR experiments were performed in triplicate.

### Buoyancy tests

The *gvpABC*, *gvpNJKFG* and *gvpVW* genes of *Anabaena* sp. PCC 7120 were amplified and cloned into pET-Duet, pET-28a and pCDFDuet-1 vectors (with different antibiotic markers), respectively. Notably, a His-tag was fused to the N-terminus of GvpA for detecting the expression of the gene cluster. Next, the three *gvp* recombinant plasmids were co-overexpressed in *E. coli* BL21 (DE3) strain. Cells were grown in liquid LB broth, induced with isopropyl β-D-1-thiogalactopyranoside (IPTG) for 4 h at 37 °C, and resuspended in 35-mm-diameter test tubes. Then, the tubes were undisturbed at room temperature for about 24 h, at which time the cell buoyancy was determined by the turbidity of the upper fraction of the culture medium. The cells transformed with the empty vectors without *gvp* gene cluster were used as the control.

### GV isolation, electron microscopy and western blot

According to a previously described protocol [18], GVs were purified from *E. coli* cells co-overexpressing the three recombinant plasmids that cover the complete *gvp* gene cluster. Carbon-coated copper grids (300-mesh) were immersed in the purified GVs for 1 min and excess liquid was removed with filter paper. GVs were negatively stained with 2% (w/v) uranyl acetate and then examined with a Tecnai G^2^ transmission electron microscopy (FEI, USA) running at 120 kV voltage. Images were taken using a CCD camera attached to the microscopy. The purified GVs were mixed with an equal volume of 2 × sample-loading buffer (100 mM Tris-HCl, pH 6.8, 4% SDS, 20% glycerol, 2% β-mercaptoethanol, 0.2% bromophenol blue), boiled for 10 min, and then applied to western blot using anti-His polyclonal antibodies.

### Cloning, expression and purification of GvpF and GvpN

The coding region of *gvpF* was amplified from the genomic DNA of *Anabaena* sp. PCC 7120, and cloned into a modified pET-29a vector with a C-terminal 6 × His-tag. The *E. coli* BL21 (DE3) strain was used for the overexpression of recombinant protein. The transformed cells were grown at 37 °C until OD_600 nm_ reached 0.8 and then induced with 0.2 mM IPTG for another 20 h at 16 °C. Cells were harvested by centrifugation (6000×g, 4 °C, 10 min) and resuspended in the lysis buffer (20 mM Tris-HCl, pH 7.5, 100 mM NaCl). After 10 min of sonication on ice and 30 min of centrifugation at 12,000×g, the supernatant was loaded onto a Ni-NTA column (GE Healthcare, Chicago, USA) equilibrated with the binding buffer, the same as the lysis buffer. The target protein was eluted with 300 mM imidazole and further applied to a Superdex 200 column (GE Healthcare, Chicago, USA) pre-equilibrated with the binding buffer. Fractions containing the target protein were collected and concentrated to 10 mg/mL for crystallization.

GvpN was expressed and purified in the same manner as GvpF. Samples for ATPase activity assays were collected at the highest peak fractions without concentration and stored at − 80 °C with 50% glycerol.

### Crystallization, data collection and structure determination

Crystals of GvpF were grown at 16 °C using the hanging drop vapor diffusion method, with a drop of 1 μL protein solution mixed with an equal volume of the reservoir solution. Crystals were obtained against the reservoir solution of 20% (w/v) polyethylene glycol 4000, 0.2 M NaCl, and 0.1 M Tris-HCl, pH 8.0. Then, they were pooled and flash cooled with liquid nitrogen after transferring to cryoprotectant (reservoir solution supplemented with 30% sucrose). The X-ray diffraction data were collected at 100 K using beamline BL17U with an EIGER X 16 M detector at the Shanghai Synchrotron Radiation Facility.

The diffraction data were indexed, integrated and scaled with HKL-2000 to the highest resolution of 2.55 Å. The structure of *M. aeruginosa* PCC 7806 GvpF (PDB code: 4QSG) was used as the search model to determine the structure of *Anabaena* sp. PCC 7120 GvpF by molecular replacement using the Molrep program [37] in the CCP4i program suite [38]. Further refinement was performed by programs REFMAC5 [39] and COOT [40]. The final model was evaluated with MolProbity [41]. Crystallographic parameters and data-collection statistics are listed in Table 1. All structure figures were prepared with PyMOL (https://pymol.org).

### ATPase activity assays of GvpN

The ATPase activity of GvpN was measured using an ATP/NADH coupled assay [42], in which the decrease of NADH is proportional to the rate of steady-state ATP hydrolysis. The reaction mixture contains 50 mM Tris-HCl, pH 8.0, 20 mM KCl, 5 mM MgCl_2_, 2.5 mM ATP, 1 mM phosphoenolpyruvate, 0.1 mM NADH, 12 U/mL pyruvate kinase (Sigma, Saint Louis, USA) and 12 U/mL lactate dehydrogenase (Sigma, Saint Louis, USA). The reaction was initiated by the addition of recombinant GvpN, final amounts of which in a 200-μL system are 0, 25, 50, 100 and 200 μg, respectively. Using a DU800 spectrophotometer (Beckman Coulter, Fullerton, USA), the decrease in absorbance at 340 nm was monitored at 25 °C at 45 s intervals for 15 min. Michaelis-Menten parameters of GvpN were calculated from the data at the concentration of NADH varying from 40 to 200 μM and in the presence of 50 μg GvpN using the Hanes-Woolf plot method.

## Data Availability

Structural coordinate of *Anabaena* sp. PCC 7120 GvpF has been deposited in the Protein Data Bank (https://www.rcsb.org/) under the accession number of 6L5D. The datasets used and/or analysed during the current study available from the corresponding author on reasonable request.

## Abbreviations

GV: Gas vesicle
*gvp* gene cluster: Gas vesicle protein gene cluster
33RR: 33-Residue repeating motif
IPTG: isopropyl β-D-1-thiogalactopyranoside
AAA protein: ATPases Associated with various cellular Activities protein.
